# Editorial: Pharmacoeconomics in the era of health technology assessment and outcomes research to prioritize resource use, innovation and investment

**DOI:** 10.3389/fphar.2023.1210002

**Published:** 2023-05-16

**Authors:** François R. Girardin, Karen Cohen, Matthias Schwenkglenks, Isabelle Durand-Zaleski

**Affiliations:** ^1^ Division of Clinical Pharmacology, Department of Laboratory Medicine and Pathology, Faculty of Medicine, Lausanne University Hospital, University of Lausanne, Lausanne, Switzerland; ^2^ Division of Clinical Pharmacology, Department of Medicine, University of Cape Town, Cape Town, South Africa; ^3^ Institute of Pharmaceutical Medicine (ECPM), University of Basel, Basel, Switzerland; ^4^ Epidemiology, Biostatistics, and Prevention Institute, University of Zurich, Zürich, Switzerland; ^5^ Université Paris Est Créteil, AP-HP URCEco Hôtel Dieu, Paris, France

**Keywords:** pharmacoeconomics, health technology assessment (HTA), patient related outcome, disinvestment choices, cost-effectiveness

Health economic evaluations in health technology assessment (HTA) focus on balancing the costs and expected benefits of interventions compared to the use of standard-of-care to leverage value to patients and healthcare payers. Pharmaco-economic (PE) studies, in particular, assess the therapeutic value of medical technologies, such as drugs or devices, and encompass multidimensional aspects, e.g., assessments of mutually exclusive drugs options, combination with pre-emptive pharmaco-genetic (PGx) testing to limit adverse reactions, or therapeutic drug monitoring as a precision medicine procedure. The extent to which findings of PE evaluations are translated into informed policy decisions depend on national healthcare systems and population expectations.

PE evaluations have the potential to streamline decision making and innovation in a wide range of therapeutic areas by determining whether the expense incurred by novel treatments is worthwhile, given the willingness-to-pay for health gains achieved, for example, measured as quality-adjusted life-years (QALY). The QALY conceptual framework was first introduced in the 1960s in studies on chronic renal failure (Klarmann et al., 1968): authors reported that the quality-of-life (QoL) with kidney transplant was 25% higher than that with dialysis. The cost per life-year gained by different therapeutic options was estimated with and without the quality adjustment. More than 50 years later, despite the inherent limitations and ethical issues, the QALY remains the most validated metric and generic measure of health to quantify the expected benefit in clinical studies.

Several instruments were developed to complement QALY metrics, such as descriptive disease-specific patient-reported outcomes (PRO), which have better face validity for clinicians. PROs provide unique insight into the outcomes of therapeutic interventions that are important to patients: they are derived from validated descriptive instruments, generic or disease-specific, which are adapted to languages and countries. The results are context specific: one should be cautious when applying them to different settings, as findings may not be transferable to other healthcare systems.

In an article on kidney transplantation, Girardin et al. analyzed the association between immunosuppressant medications and the QoL outcomes in 558 kidney transplant recipients in France, Germany, Spain, and Switzerland. VAS scores and EQ-5D utility scores were adjusted for patient characteristics and medical history. Both elicitation instruments delivered sound results for QoL in kidney transplant patients. Most patients received tacrolimus and mycophenolate mofetil in all four countries. During one-year of follow-up, a significant proportion of patients switched immunosuppressive therapy (according to country, from 20% to 40%), which was associated with worse QoL, irrespective of the initial medications. Although initial treatments were comparable, patient characteristics and evolving trends differed across countries more than between centers.

Several articles in our Research Topic address Research Topic in oncology, where emerging, expensive therapies have received much attention in recent years. Concerns on the clinical side include market entries on the basis of immature data on hard patient outcomes, most importantly overall survival (Prasad et al., 2015) (Paoletti et al., 2020), but also limited understanding of patient perceptions. Incorporating PROs in drug labels has been proposed as a means of giving more weight to cancer patient perspectives in regulatory decisions. A review of Food and Drug Administration (FDA) and European Medicines Agency (EMA) oncology drug labels by Cella et al. revealed relevant limitations and inconsistencies with respect to PRO inclusion, even potential biases towards positive outcomes. This indicates a need for improved and more harmonized guidance, to better inform drug prescribers and users.

Budget constraints are a common issue in most healthcare systems. While the prices of new oncology drugs are matter of growing concern even in high income countries (Godman et al., 2021), low- and middle-income countries, struggle with the costs of oncology drugs despite international price differentials (Al-Ziftawi et al., 2021). These issues are aggravated by restricted population access due to lack of universal healthcare coverage. Locally developed drugs may in some cases contribute to affordability, thus easing the economic burden on patients and their families. The work of You et al. exemplified this concern: the authors found that adebrelimab, an immune checkpoint inhibitor (ICI) developed in China as IgG4 monoclonal antibody against PD-L1, may be a cost-effective option for first-line treatment of extensive-stage small cell lung cancer, from the perspective of the Chinese healthcare system, even though earlier studies found other ICI not to be cost-effective in this indication in China. Lack of cost-effectiveness, partially driven by high drug prices and in some cases limited value for patients (Pontes et al., 2020), also occurs in industrialized countries. In the US study, Li et al. concluded that nivolumab (another ICI) is not cost-effective compared to sorafenib as a first-line therapy for advanced hepatocellular carcinoma. There were hints at cost-effectiveness differences between patient subgroups, specifically in patients with intermediate-stage disease (Barcelona Clinic Liver Cancer stage B).

Heart failure (HF) is an increasing health concern that imposes high costs and resource use. HF management stems from the use of highly cost-effective angiotensin converting enzyme inhibitors (ACEi) and β-blockers to the use of novel medication targets, such as ivabradine, vericiguat, or sodium-glucose cotransporter-2 inhibitors (SGLT2i) dapagliflozin and empagliflozin. Lim et al. reviewed pharmacoeconomic and cost-effectiveness studies of SGLT2i, ARNi, ivabradine, vericiguat, and omecamtiv. Pharmaco-economic analyses of empagliflozin in HF patients with Type 2 diabetes and dapagliflozin for HF with reduced ejection fraction remained below the willingness-to-pay thresholds in most middle- and high-income countries. Still, vericiguat was found cost effective at a higher cost per QALY threshold than SGLT2i. The authors concluded that although cost-effectiveness on newer medications, such as SGLT2i, ARNi, ivabradine, vericiguat, and omecamtiv in HF with reduced ejection fraction is established, there is still lower evidence for their use in HF with preserved ejection fraction that accounts for the majority of HF. Eventually, in low- and middle-income countries, the fundamental recommendation would be that patients be diagnosed early and treated with multiple, sourced renin-angiotensin drugs that remain highly effective and inexpensive rather than with expensive and *a priori* cutting-edge drugs.

Effectiveness and cost-effectiveness evaluation may be particularly challenging when pre-emptive measures are considered, such as pharmacogenomics (PGx) applied to prevent gene-drug related adverse reactions. Van der Wouden et al. found that nation-wide adoption of PGx-guided initial dose and medication selection of single actionable drug-gene interactions could potentially avoid fatal outcomes in 0.3% of patients taking medications such as clopidogrel, capecitabine, 5-FU, thiopurines or irinotecan: the expected cost would be €51000 per prevented death. Still, the evaluation of surrogate endpoints due to wrong drug selections or dosages remain a complex process that must be validated by probabilistic approaches and sophisticated statistic frameworks to address strong assumptions and uncertainty (Buyse et al., 2016; Ciani et al., 2022). Despite no manuscript was submitted in this field, gene therapies for patients with orphan diseases remain a key concern when developing cost-effectiveness decision-models, given the uncertainty in several situations and the enormous pressures put on health authorities to fund any new medicine in this area despite high prices (Luzzatto et al., 2018).

Ninomyia et al. explored a PGx-informed clozapine therapy and blood monitoring schedule based on novel SLCO1B3-SCLO1B7 variants in addition to HLA variants to leverage genotyping test sensitivity for the detection of clozapine-induced agranulocytosis and granulocytopenia (CIAG). By adding SLCO variants, the expected test sensitivity increased, whereas the specificity decreased (89.0%–86.9%) still increasing the overall risk predictability (Ninomiya et al.). Incorporating new SLCO variants to pre-emptively assess CIAG risk improved the effectiveness of PGx-guided clozapine administration: SNP-based predictive tests differ between ancestral groups due to alleles or haplotypes frequencies and varying patterns of linkage disequilibrium (Islam et al., 2022).

New diagnostic tests and drugs are being developed and tested as shown in the clinical articles published in this issue. They offer additional health benefits, which often require additional healthcare resources to be committed at a certain cost (Darlington et al.). To ensure patient access to these novel medications and diagnostic technologies, and to secure the sustainability of healthcare systems, several routes are explored. In addition to increasing the resources committed to healthcare (which is happening in most countries), reducing unnecessary care, and considering decremental cost-effective strategies are current options. The use of PGx allows to reduce risks as shown in two articles published in this issue: the implementation of molecular diagnostics should better identify the most suitable target populations for drugs and reduce overuse. A step further is the consideration of decremental cost-effective strategies. These become relevant in situations where a small health loss can be acceptable in exchange for a large monetary gain reallocated to the healthcare system. Methodologically, related studies follow similar principles as non-inferiority studies, which define an inferior margin of difference, yet acceptable, for therapeutic innovations *versus* the standard-of-care. The development of non-inferiority studies in recent years offers a range of possibilities for economic studies that would identify areas for disinvestment. However, transforming those studies into policies necessitates reassurance that the money saved will be efficiently used for the provision of healthcare. It might also be necessary to provide financial incentives to both health professionals and patients to overcome resistance to change or loss of revenue. For instance, different types of incentives are currently in place to limit the resource use related to medications, with positive incitements for physicians, pharmacists, and patients to foster the use of generic drugs or biosimilars.

Ultimately, HTA and PE could be considered foundation not only for outcome research, but also for comparative research regarding future innovations and investments in the development of precision medicine and personalized therapies.

The editors of the Research Topic would like to thank all the participating contributors for their valuable involvement in the success of the issue on HTA and PE.
